# Prediction and Prioritization of Rare Oncogenic Mutations in the Cancer Kinome Using Novel Features and Multiple Classifiers

**DOI:** 10.1371/journal.pcbi.1003545

**Published:** 2014-04-17

**Authors:** ManChon U, Eric Talevich, Samiksha Katiyar, Khaled Rasheed, Natarajan Kannan

**Affiliations:** 1 Department of Computer Science, University of Georgia, Athens, Georgia, United States of America; 2 Department of Dermatology, University of California San Francisco, San Francisco, California, United States of America; 3 Department of Biochemistry and Molecular Biology, University of Georgia, Athens, Georgia, United States of America; 4 Institute of Bioinformatics, University of Georgia, Athens, Georgia, United States of America; Indiana University, United States of America

## Abstract

Cancer is a genetic disease that develops through a series of somatic mutations, a subset of which drive cancer progression. Although cancer genome sequencing studies are beginning to reveal the mutational patterns of genes in various cancers, identifying the small subset of “causative” mutations from the large subset of “non-causative” mutations, which accumulate as a consequence of the disease, is a challenge. In this article, we present an effective machine learning approach for identifying cancer-associated mutations in human protein kinases, a class of signaling proteins known to be frequently mutated in human cancers. We evaluate the performance of 11 well known supervised learners and show that a multiple-classifier approach, which combines the performances of individual learners, significantly improves the classification of known cancer-associated mutations. We introduce several novel features related specifically to structural and functional characteristics of protein kinases and find that the level of conservation of the mutated residue at specific evolutionary depths is an important predictor of oncogenic effect. We consolidate the novel features and the multiple-classifier approach to prioritize and experimentally test a set of rare unconfirmed mutations in the epidermal growth factor receptor tyrosine kinase (EGFR). Our studies identify T725M and L861R as rare cancer-associated mutations inasmuch as these mutations increase EGFR activity in the absence of the activating EGF ligand in cell-based assays.

## Author Summary

Cancer progresses by accumulation of mutations in a subset of genes that confer growth advantage. The 518 protein kinase genes encoded in the human genome, collectively called the kinome, represent one of the largest families of oncogenes. Targeted sequencing studies of many different cancers have shown that the mutational landscape comprises both cancer-causing “driver” mutations and harmless “passenger” mutations. While the frequent recurrence of some driver mutations in human cancers helps distinguish them from the large number of passenger mutations, a significant challenge is to identify the rare “driver” mutations that are less frequently observed in patient samples and yet are causative. Here we combine computational and experimental approaches to identify rare cancer-associated mutations in Epidermal Growth Factor receptor kinase (EGFR), a signaling protein frequently mutated in cancers. Specifically, we evaluate a novel multiple-classifier approach and features specific to the protein kinase super-family in distinguishing known cancer-associated mutations from benign mutations. We then apply the multiple classifier to identify and test the functional impact of rare cancer-associated mutations in EGFR. We report, for the first time, that the EGFR mutations T725M and L861R, which are infrequently observed in cancers, constitutively activate EGFR in a manner analogous to the frequently observed driver mutations.

## Introduction

Cancer is a complex disease in which healthy cells undergo a series of genetic changes, eventually becoming cancerous, growing uncontrollably and spreading throughout the body [1]. Identification of the specific genetic changes that promote cancer traits within a cell can yield clues into potential treatments for the disease. Large-scale cancer genome sequencing studies have thus been initiated in order to catalog the mutations observed in human cancers [2]–[6].

Not all mutations have equal influence on the disease state of a cell, however. Certain mutations, called “drivers,” are known to have a causative effect, driving the transformation of a cell from healthy to cancerous, often by promoting cell growth or inhibiting apoptosis (programmed cell death) [1]. In contrast, the majority of mutations do not significantly affect the cancer characteristics of a cell, and can be considered relatively benign “passengers” in a tumor cell [7]. Mutated driver genes are worthwhile targets for drug discovery, because counteracting the mutation's effects can potentially slow or reverse cancer progression in individual patients [8], [9]. To fully realize this potential, however, there is a need to develop computational approaches that can (i) distinguish causative from non-causative mutations, and (ii) identify key causative mutations for experimental studies and clinical targeting.

Indeed, several previous studies have proposed methods to predict causative mutations in cancer genomes (reviewed in [6]). These methods fall into three major categories: (i) frequency-based approaches, (ii) structure-based methods and (iii) statistical and machine learning methods. Frequency-based approaches are based on the assumption that the mutations that occur in multiple patient samples are likely to be those that are causative [10]. Although such assumptions are valid for some recurrent mutations such as the L858R mutation in epidermal growth factor receptor (EGFR) in lung cancer [11] and V600E in BRAF in melanoma [12], [13], there is emerging evidence that rare mutations can be drivers [7], [14]–[16]. Moreover, a comprehensive analysis of several breast and colorectal cancer genomes revealed that the genomic landscapes of these cancers are dominated by a large number of rare gene mutations rather than recurrent oncogene mutations [17].

Structure-based methods offer a powerful way of predicting the impact of mutations by taking into account the three-dimensional context of the mutated residues [18]–[21]. However, such approaches are not applicable on a genome-wide scale because of the lack of experimental structure information for several oncogenes.

Because a wide variety of factors play into the oncogenic effect of any given mutation, machine learning approaches have become a method of choice to predict causative mutations based on a variety of contextual information [18], [22]–[30]. In general, supervised machine learning approaches learn from the features of known cancer-associated and benign mutations to classify unknown mutations. (Note that the term “cancer-associated” refers to mutations that are predicted to have direct or indirect oncogenic effect, while the term “driver” refers to confirmed causative mutations.) Although several machine learning-based methods have been proposed previously, there still remains a need to improve the sensitivity and efficacy of existing methods [28]. For example, most existing approaches use standard features of mutated residues for training the classifier and do not take into account gene- or family-specific features that can improve prediction accuracy [28]. Furthermore, existing approaches use one or two common machine learning algorithms and do not consider the biases introduced by these algorithms. Finally, existing approaches typically provide a binary “yes” or “no” classification for disease association, which does not solve the problem of prioritizing candidate mutations for follow-up experimental studies [31].

Here we apply a novel machine learning approach to predict and prioritize cancer-associated mutations in protein kinases, a class of proto-oncogenes frequently mutated in human cancers. Our approach differs in three major ways from previous approaches. First, we introduce new kinase-specific features, beyond those used in previous methods [28],[32],[33], to improve prediction accuracy. Mutations in the kinase domain, and particularly those at functional sites, have been shown to be more likely to be oncogenic [34], typically through mechanisms that constitutively activate the kinase [35]. Second, we use a multiple classifier approach (ensemble method), which by combining multiple machine learning algorithms (individual classifiers), overcomes the biases introduced by each method. Finally, we use our combined classifier to produce a numerical ranking of cancer-associated mutations in EGFR and test the impact of predicted mutations on EGFR kinase activity using cell-based assays. Our studies identify T725M and L861R as rare cancer-associated mutations in that EGFR kinase harboring these mutations display constitutive kinase activity.

## Methods

### Data sources

The data sources used for training and evaluating the classifiers consist of a “positive” set of known cancer-associated mutations, a “negative” set of known benign mutations, and several attributes of proteins and amino acids, which we drew from several databases. The parameter settings used and detailed results for construction and evaluation of each classifier are described in detail in Supplementary Text S1.

#### Positive mutation set: Oncogenic mutations

As the basis for our “positive” set we used the Catalogue of Somatic Mutations in Cancer (COSMIC), a database of somatic mutations observed in cancer samples gathered from published literature sources and provided by the Wellcome Trust Sanger Institute's Cancer Genome Project (CGP) [2], [36]. The mutations from the COSMIC database (versions 50 and 57) were each filtered by gene name for human protein kinases as identified in KinBase [37]. We then filtered these mutations for those occurring in the protein kinase domain, as identified by ProKinO [38], yielding 1451 distinct point mutations in 224 protein kinase genes. Each kinase gene was therefore associated with several mutations; EGFR kinase, for example, has 278 mutations in the protein kinase domain.

As COSMIC is a catalog of somatic mutations observed in clinical samples, it potentially contains both causative and non-causative mutations. Somatic mutations cover a small portion of the overall genome; it is thus relatively rare that the same somatic mutation occurs in two or more patient samples unless the mutation is associated with the patients' shared phenotype, in this case cancer. We therefore selected a subset of mutations which have been observed more than once in COSMIC, as an initial list of 226 likely causative mutations to use as the “positive” (disease) set in our classification.

#### Negative mutation set: Benign polymorphisms

The “negative” (benign, non-disease) set consisted of the 331 non-synonymous mutations in protein kinase genes obtained from SNP@Domain, an online database of naturally occurring single nucleotide polymorphisms (SNPs) within protein domain structures and sequences [39], which was itself originally derived from dbSNP [40]. We consider commonly occurring polymorphisms to be effectively benign because the polymorphisms were originally sampled from healthy individuals, an approach previously used by others (e.g. [26]).

#### Protein features

Features of the mutations used for training the classifiers were retrieved from several sources. The hierarchical classification of protein kinases into major groups and families is according to KinBase [37]. Amino acid properties, such as hydrophobicity and molecular weight, were taken as listed in the data files included with EMBOSS [41]. Protein sequence annotations were retrieved from the UniProtKB database [42]. We also used protein sequences from the UniRef90 database [43] in our own calculations of amino acid conservation. Position of mutation in the kinase domain and subdomain location of the mutation were also used (see section “Feature preprocessing” below).

Of the 518 protein kinases in the human genome, several are “atypical” kinases which lack sequence similarity to the other kinases. Thus, certain features could not be calculated for the atypical kinases, leaving 503 distinct protein kinase genes for which we successfully extracted features for machine learning.

### Feature preprocessing

Mutations are uniquely identified by the gene name, protein sequence position, wild-type amino acid, and mutant amino acid type. We extract features related to biochemical, structural, functional and evolutionary properties, which in the end generated 29 features in total, as follows.

Of these 29 features, 23 were previously explored by others, including amino acid biochemical properties, sequence conservation, and kinase subdomain [25], [27], [44], [45]. Our novel features are the protein kinase classification terms (group and family) and the conservation levels of the wild type and consensus type within alignments of all, group– and family-specific kinase sequences.

#### Comparative

The protein kinase superfamily constitutes a diverse range of enzymes which can be classified hierarchically into 8 major groups (plus the “other” and “atypical” categories) and multiple families within each group, each with distinct mechanisms and interacting partners [46]. To account for these potential differences, we tracked the names of each kinase's group and family, per KinBase. Additionally, we calculated the evolutionary conservation of the wild-type or consensus amino acid type among diverse eukaryotic species at each position within the protein kinase domain. This approach considers the evolutionary process as “nature's laboratory”: over the course of over 2 billion years since the divergence of eukaryotes, each type of mutation at each position in the protein kinase gene has occurred many times, simply by chance as part of a random mutational process. The mutations that do not alter the overall functioning of the protein are tolerated, and are likely to be observed in extant species, while substantially deleterious mutations are more likely to be eliminated from the gene pool of surviving organisms.

Since the association between evolutionary conservation and disease-causing mutations in protein kinases is not yet fully understood, we calculated conservation at three evolutionary depths: within the same PK family (close evolutionary relatives), within the same PK major group (greater evolutionary distance), and among all eukaryotic protein kinases (ancient divergence, preserving only the overall fold of the protein structure). While conservation of amino acid types has been considered in previous studies, our distinction between depths of divergence at the family and group levels is novel. In addition, we calculate conservation relative to both the wild-type residue and the “consensus” residue type in an alignment. The conservation value at a given alignment site is the frequency of the wild-type or consensus-type residue in the alignment column. The calculations are performed on large multiple-sequence alignments using sequences from UniRef90 and aligned using the MAPGAPS program [47] and HMMer 3.0 [48].

#### Amino acid properties

These features describe the properties of individual amino acids in the sequence of both the healthy, normally occurring “wild-type” protein and the mutant, as well as the difference between the two where this can be quantified. The properties are the hydropathy index (hydrophobicity), charge, polarity, van der Waals volume, molecular mass, and naturally occurring residue frequency. We also include the BLOSUM62 amino acid substitution matrix values [49] as a measure of the overall similarity between the wild-type and mutant residues.

#### Structural and functional

We introduced features that captured the structural and functional location of mutations. The eukaryotic protein kinases can be identified by a set of 11 structural regions, known as subdomains, which are conserved across the entire superfamily [50]. We identified the sequence locations of these subdomains in each of the 503 typical protein kinases in the human genome, using a Gibbs motif sampler with curated motif models for each subdomain, similar to the method described in [28]. Mapping the position of each mutation onto these regions allowed us to identify which sub-domain the mutation belongs to, if any. Two additional features describe functional roles of amino acids: whether a position is a binding site (e.g. for ATP or another protein), and the post-translational modification of the residue, if any (e.g. phosphorylation, which has been shown to have a significant effect on protein stability and function [51]).

### Feature selection

Feature selection serves two purposes: to choose a smaller, more computationally tractable subset of meaningful features which can be used to effectively predict the target attribute (causative or non-causative), and to understand the relative usefulness or contribution of each feature toward predicting the target [52]. With emphasis on the latter, we independently applied five feature selection algorithms, namely OneR algorithm [53], relief-based selection [54], chi-square selection, a gain-ratio-based filter approach [55] and correlation-based selection [56], to evaluate our attributes.

Evaluation was performed on the combined positive and negative data sets, with the positive set also including COSMIC mutations that occur only once, in order to obtain a larger data set for this step. The detailed feature selection results obtained from the five selection methods with 10-fold cross-validation are given in Table S2. 

We used the 10-fold cross-validation routine implemented in Weka [57] to select the most relevant features from all the attributes we considered. As with other parts of the learning cycle, 10-fold cross-validation randomly splits the data up into 10 disjoint subsets. However, in the feature selection evaluation routine only the training folds are used, and there is no testing as such [58]. Feature selection is run on each training fold (90% of the data) in turn and the results are summarized. In the case of single-attribute evaluators (in our case OneR, Chi-Square, Relief, and Gain-Ratio), the output shows the average merit and average rank of each attribute over the 10 folds along with their respective standard deviations. In the case of subset evaluators such as Correlation-based feature selection in our case, the output shows, for each attribute, in how many folds it was part of the final best subset selected. In both these cases the aim is to provide some measure of robustness as well as stability of the feature with respect to small changes in the distribution of input attribute values.

The attributes were ranked in terms of effectiveness as a predictor according to each selection method. Those attributes selected by at least 3 out of 5 (60%) of the selection methods were retained, yielding a final feature subset of 17 selected features (from the original 29) which we used as input for the training process in subsequent analysis (Table 1). We also determined a score indicating the usefulness of each selected feature by taking the arithmetic mean of the feature's ordinal ranking across all 5 selection algorithms. This “average rank” score enables a complete ranking of the selected features, with scores ranging from 1.4 to 13 (Table 1).

**Table 1:** Selected protein features.

Feature	Votes	AvgRank
Protein kinase family	5	1.40
Protein kinase group	5	1.80
Amino acid type, WT	5	8.00
BLOSUM62 pairwise score	5	8.20
Side-chain polarity, mutant	5	11.00
Conservation of wild type in all kinases	5	11.60
Conservation of consensus type in kinase group	5	11.60
Conservation of consensus type in all kinases	5	13.00
Conservation of consensus type in kinase family	4	5.75
Kinase subdomain	4	6.00
Average mass of amino acid, WT	4	7.50
Is a binding site?	4	8.25
Van der Waals volume, WT	4	8.75
Site modification type (if any)	4	9.25
Amino acid type, mutant	4	10.75
Side-chain polarity, WT	4	11.50
Is in protein kinase domain?	3	11.67

The “Votes” column indicates how many feature selection algorithms cast a vote for that particular feature during the 10-fold cross-validation selecting procedure; the “Avg Rank” column describes the averaged rank of a particular feature within the selected algorithms.

### Machine learning methods

To classify point mutations in human protein kinase sequences as either cancer-associated or non-causative, we applied 11 machine learning methods to our dataset. The machine learning methods are J48 (Tree) [59], [60], Random Forest [61], NB Tree [62], Functional Tree [63], Decision Table [64], DTNB [65], LWL (J48+KNN) [66], Bayes Net [67], Naive Bayes [68], SVM [69], [70], and Neural Network [71].

The detailed results of evaluation of each classifier as well as several alternative approaches are described in Supplementary Text S1.

#### Cross-validation

To account for the possibility of over-fitting or bias resulting from small sample sizes, we applied 10-fold cross-validation [72], [73] to train all of the models presented in this article. Cross-validation is a robust approach to validate the training methods as it repeatedly tests different trained models on “unseen” data [72]. In 10-fold cross-validation, the training set is randomly split into 10 equal subsets (folds) and the algorithm is repeatedly trained on nine subsets and tested on the remaining subset, which is a set of “unseen” data. This ensures each instance is included into the testing set once. The final results are the average of the 10 independent training models.

#### Evaluation

We compared the performance of the 11 machine learning algorithms in classifying the mutations in the positive and negative set. Due to the highly imbalanced dataset in certain experiments, a meta-learner, Cost Sensitive Classifier, is used to define the confusion matrix to optimize the learning process [74].

We evaluated the performance of the classifiers in terms of accuracy, precision and F-measure (or F1 score), a measurement index which is more robust to highly imbalanced datasets than accuracy [55]. The F-measure is defined as a harmonic mean of precision and recall:
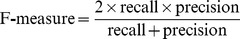
where precision, or positive predictive value, is the proportion of true positives in the accepted set (TP/(TP+FP)) and recall, also known as sensitivity or true positive rate, is the proportion of all positives that were included in the accepted set (TP/(TP+FN)).

### Combining multiple classifiers to prioritize EGFR mutations

Having evaluated the 11 trained models that are described in the previous section, we selected the models trained on the combined well-performing positive sets — mutations that appear more than once in the COSMIC dataset — for further application. We focused on the gene EGFR, a protein kinase that is frequently mutated in lung cancer, and used the previously trained models to evaluate the EGFR mutations that appear only once in the COSMIC dataset, as these mutations were excluded from the initial training set. Since these mutations have not been replicated in other tumor samples, it is more likely that some of them are not significantly associated with cancer. Thus, we use the following approach to combine the predictions of the trained classifiers to sort these EGFR mutations by their likelihood to be cancer-associated.

For each of the non-synonymous point mutations in the kinase domain of EGFR that were observed only once in COSMIC, we calculated a numerical score for the likelihood of a given mutation to be cancer-associated using two different approaches: a simple majority voting approach with one “vote” per classifier, and a more sophisticated approach in which each classifier's “vote” is weighted by its accuracy as previously estimated by cross-validation. Mutations that have been classified as cancer-associated by more classifiers are considered more likely to be true positives, whereas fewer “votes” indicate a mutation is less likely to be cancer-associated.

#### Majority voting approach

In this approach, each of the 11 trained classifiers predicts whether a given mutation is cancer-associated, and a positive prediction is counted as one “vote”. The votes are then counted to produce a score in the range of 0 to 11:
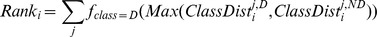
in which *i* is the number of instances, *j* is the number of classifiers, *D* is disease, *ND* is non-disease, function 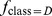 equal to 1 if the Max classification distribution is disease (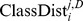). To distinguish between cancer-associated and benign mutations with this score, we chose a simple majority-vote cutoff (i.e. 6 or more votes is treated as a positive prediction).

#### Weighted voting approach

The majority ranking approach places equal weight on the predictions made by each of the 11 classifiers. To account for the differences in predictive ability between individual classifiers, we produced an alternative ranking in which each classifier's “vote” is weighted by its accuracy (as determined previously by 10-fold cross-validation). The vote here is the sum of the probability of instance *i* being classified as causative, weighted by the accuracy of corresponding classifier. The sum is then normalized by dividing by the sum of the accuracies of the 11 classifiers
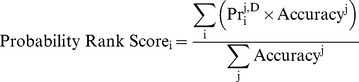
The result is a decimal score between 0 and 1 where values above 0.5 indicate a positive prediction.

### Experimental characterization of EGFR mutations

#### Mutational analysis, transfection and immunoblotting

To test the impact of mutations, CHO cells were transiently transfected with GFP-tagged WT or mutant-EGFR plasmids (pEGFP-N1-EGFR plasmid was a kind gift from Dr. Graham Carpenter). Point mutations were generated by using Quik Change II site-directed mutagenesis kit (Stratagene) and confirmed by DNA sequencing. Cells were grown in high-glucose Dubecco's Modified Eagle Medium (DMEM) (Cellgro, Manassas, VA, USA) with 10% fetal bovine serum (Bioexpress, UT, USA) without antibiotics. Transient transfection was performed using lipofectamine-2000 (Invitrogen, Carlsbad, CA) according to manufacturers protocol with wild type and mutant EGFR.

To detect auto-phosphorylation of WT/mutant EGFR, transient transfected cells were serum starved in Hams F-12 media for 18 hours. EGF stimulation was carried out using 100 ng/mL human EGF (Sigma, St Louis, MO) for five minutes. Cells were washed with 1× PBS and immediately lysed in lysis buffer (50 mM Tris-HCL, pH 7.4, 150 mM NaCl, 10% glycerol, 1 mM EDTA, 10% Triton X-100, 1 mM PMSF, and 1× Protease Inhibitor Cocktail Set V, EDTA-free). Western blotting was done using GFP, pY1173, pY1068, pY845, pY1045, pY1086 (Cell Signaling, Danvers, MA), and tubulin (Millipore, Billerica, MA) antibodies.

## Results

### Conservation and kinase-specific features contribute to the classification of cancer-associated mutations

We first sought to determine a subset of features that show high predictive value in distinguishing cancer-associated from benign mutations, and to evaluate the contribution of the kinase-specific features we introduced in this study, namely the hierarchical protein kinase classification levels (group, family) and the conservation levels at each evolutionary depth (all kinases, group and family).

We applied 5 different feature selection algorithms (see Methods), each of which selected a subset of the full feature set, to produce a ranking of the 29 features, 17 of which met our criteria for inclusion in the final feature subset used for training the classifiers (Table 1). All 5 selection algorithms selected the features “Protein kinase family” and “Protein kinase group”, and each individual algorithm ranked these two features at the top. The features “amino acid type (WT)”, “BLOSUM62 score”, and “side chain polarity (Mutant)” were also selected by all 5 algorithms and ranked highly by individual algorithms. Conservation scores of the wild-type residue among all kinases, and of the alignment consensus type among all kinases, among the major groups and among major families, were also ranked highly, indicating that they extensively contribute to the prediction of the target attribute, a result that supports the importance of our novel proposed features.

To further test the contribution of the new features, we re-ran our classification experiments after removing the novel kinase-specific features from the 17 features identified through the feature selection process. Notably, the performance (as indicated by accuracy values in Supplementary Dataset S1) reduces substantially when the kinase-specific features are removed. We then performed a chi-squared test of the number of correct and incorrect predictions made by each of these two classifiers on the 557 mutations in our final training set: 551 correct and 6 incorrect versus 498 and 59 for the full and reduced feature sets, respectively. This statistical test confirmed that the decrease in accuracy when the novel features are removed is significant at *p*<.001 (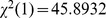, 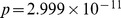, simulated 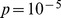 based on 100,000 replicates).

It is also interesting to note that the performance of the combined classifier is much less degraded when the kinase-specific features were removed compared to the single classifier (Dataset S1), suggesting that the multiple classifier approach contributes to stability and robustness.

### Combining multiple classifiers improves the prediction of cancer-associated mutations

We used a weighted voting approach to combine 11 single classifiers to be a more robust ensemble classifier.

Table 2 and Table 3 present the *in silico* experimental results in terms of confusion matrix and several other measurement indexes which quantify the performance of the individual classifiers. All 11 classifiers performed fairly well, with recall rates at least 95.6% and False Positive (FP) rates at most 10.6% (Table 3). Of the individual classifiers, SVM performed the best on most metrics. However, the combined classifier performs better than the individual classifiers, reaching 98.7% for both precision and recall.

**Table 2:** Confusion matrix of individual classifier performance.

Algorithms	TP	FN	TN	FP
J48 (Tree)	221	5	318	13
Random Forest	216	10	320	11
NB Tree	217	9	311	20
Functional Tree	217	9	323	8
Decision Table	222	4	296	35
DTNB	219	7	321	10
LWL(J48+KNN)	220	6	316	15
Bayes Net	221	5	313	18
Naive Bayes	218	8	309	22
SVM	219	7	323	8
Neural Network	218	8	321	10
Combined (0.5)	223	3	328	3

All *in silico* experiments were evaluated with 10-fold cross-validation. TP means an instance in the positive set (COSMIC) was correctly classified as causative, TN means an instance in the negative set (dbSNP) was correctly classified as non-causative.

**Table 3:** Comparison of performance of individual and combined classifiers.

Algorithm	TP Rate	FP Rate	Accuracy	Precision	Recall	F-Measure
J48 (Tree)	0.978	0.039	0.968	0.944	0.978	0.961
Random Forest	0.956	0.033	0.962	0.952	0.956	0.954
NB Tree	0.960	0.060	0.948	0.916	0.960	0.937
Functional Tree	0.960	**0.024**	0.969	0.964	0.960	0.962
Decision Table	**0.982**	0.106	0.930	0.864	**0.982**	0.919
DTNB	0.969	0.030	0.969	0.956	0.969	0.963
LWL(J48+KNN)	0.973	0.045	0.962	0.936	0.973	0.954
Bayes Net	0.978	0.054	0.959	0.925	0.978	0.951
Naive Bayes	0.965	0.066	0.946	0.908	0.965	0.936
SVM	0.969	**0.024**	**0.973**	**0.965**	0.969	**0.967**
Neural Network	0.965	0.030	0.968	0.956	0.965	0.960
Combined (0.5)	**0.987**	**0.009**	**0.989**	**0.987**	**0.987**	**0.987**

Each algorithm trained using selected features and evaluated with 10-fold cross-validation. Values are average of the metrics evaluated with respect to the positive and negative classes.

An alternative metric is the “F-Measure”, a harmonic mean of precision and recall, on which all single classifiers achieved a score of at least 0.919 (Table 3), a result consistent with previous studies [27]. The high F-measure score of 0.989 for the combined classifier also vouches for the stability of our feature set on the relatively small training dataset. Furthermore, the competitive performance of the 11 single classifiers suggests that they each contribute to the improved performance of the multiple (ensemble) classifier.

We used two separate 10-fold cross-validation loops, one for feature selection and another for training and testing. Using the cross-validation terminology described in [58], our approach is considered an OUT method (in which feature selection is done outside the training/testing loop) rather than an IN method (in which feature selection is in the same loop as training and testing). This may have caused a problem called “information leak” [58] due to the fact that the full data set was exposed to the feature selection methods before the training/testing cross-validation loop. However, the potential information leak is partially compensated for by the robust and comprehensive approach we used for feature selection, using multiple feature selection methods and cross-validation loops for each of them (see Methods). In the design of this study, it was necessary to use a single, fixed set of features for both supervised and unsupervised learning (discussed below). Furthermore, the OUT method does not affect the relative performance of the different classifiers [58], which is more important in this study than the absolute accuracy of each classifier. Nevertheless, the performance evaluation of the 11 single classifiers and the combined classifier shown in Table 3 should be interpreted with caution given the potential for bias due to information leak.

### Validation of positive and negative sets by combined supervised and unsupervised clustering

Since there exists a level of uncertainty in the labels (“cancer-associated” and “benign”) in our dataset, the predictive model that is trained by the supervised learning approach, resulting in the combined classifier, might be biased. In this section, we denote the prediction of the combined classifier as the Supervised score (S-Score). We introduce another unsupervised learning module to help reduce the label uncertainty. The unsupervised module performs clustering using Euclidean distance in the space of the 17 selected features, without considering the labels, and the labels are only used for the computation of the Unsupervised Score (U-Score), which measures cancer-association based on clustering in the feature space. We conducted further analysis on our dataset by combining and comparing both S-Score and U-Score (see section “Learning Methods” in Supplementary Text S1) because such comparisons can potentially reveal suspicious mutations labeled incorrectly. Mutations with both S-Score and U-Score above 0.5 are considered cancer-associated while mutations with both U and S-scores below 0.5 are considered benign. All other mutations are considered uncertain, or suspicious.

Our analysis reveals that majority of cancer-associated and benign mutations fall into “Expected” clusters. Specifically, 219 out of 226 instances (≈97%) labeled as cancer-associated fall into the “Expected” category, and 255 out of 331 instances (≈77%) labeled as benign fall into the “Expected” category (see section “Identifying Suspicious Mutations in COSMIC-FG1 v.57” in Supplementary Text S1).

### Application of an ensemble classifier to predict rare variants in EGFR

We combined the 11 classifiers to effectively identify and prioritize rare EGFR mutations for experimental studies. We ranked the unconfirmed mutations in EGFR using the combined classifier (Table 4). The detailed results and log files of the computational experiments are given in Table S3.

**Table 4:** Top predicted unconfirmed mutations.

Rank	Priority Score	Position	WT	Mutant
1*	0.97699	861	L	R
2*	0.97649	724	G	S
3	0.97644	721	G	S
4	0.97577	858	L	K
5	0.97566	721	G	D
6	0.97559	861	L	P
7	0.97558	862	L	P
8	0.97509	719	G	A
9	0.97507	721	G	A
10	0.97483	729	G	R
11	0.97369	857	G	E
12	0.97365	719	G	V
13	0.97185	854	T	A
14	0.97110	735	G	S
15	0.97023	856	F	S
16	0.96854	856	F	L
17	0.96507	729	G	E
18	0.96462	855	D	G
19	0.96399	779	G	S
20	0.96291	858	L	A
21*	0.96238	725	T	M
22	0.96210	858	L	W
23	0.96034	779	G	C
24	0.95998	723	F	S
25*	0.95649	858	L	Q
26	0.95400	858	L	M
27	0.95381	731	W	R
28	0.95333	799	L	R
29	0.95268	720	S	P
30	0.95253	838	L	P
…				
161*	0.61788	746	E	K

Probability scores and rankings of the top predicted mutations. Scores were calculated with the multiple classifier trained on COSMIC v.50 data. Asterisks indicate the five mutations selected for cell-based assays.

#### Selection of mutations for *in vitro* experiments

Based on our prioritization as ranked by the weighted voting approach, we selected four mutations from the top 30 ranked mutations for further investigation. The selected mutations are L861R and G724S (ranked #1 and #2), and two slightly lower-ranked sites, T725M (ranked #21) and L858Q (ranked #25). Additionally, we selected a fifth mutation that was not strongly predicted to be either cancer-associated or benign, E746K (ranked #161). The choice of lower-ranked mutations for experimental studies was based on a variety of factors including crystal structure analysis, as described below.

#### Rare variants identified as potentially causative

**Figure 1:**
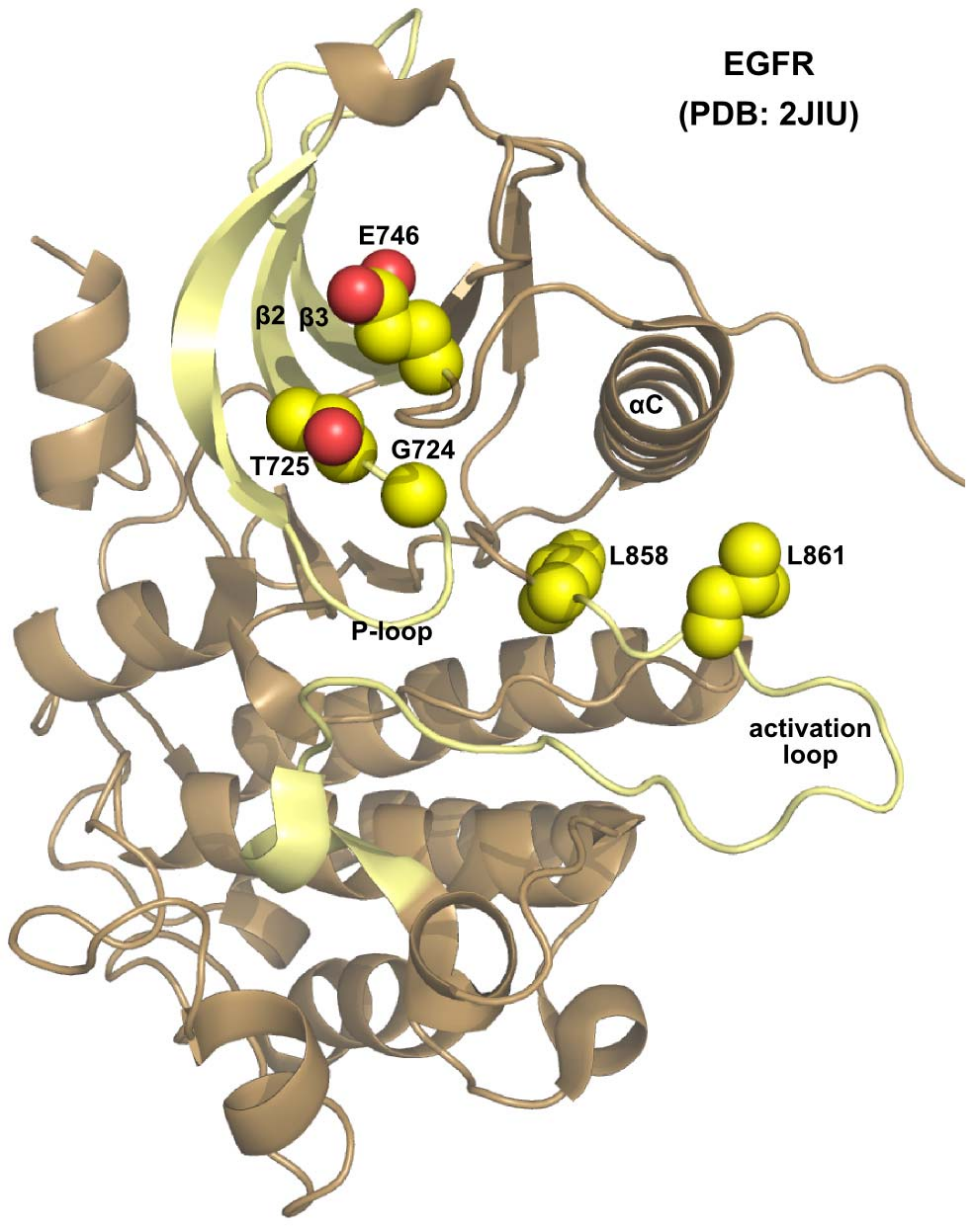
Structural location of selected EGFR mutation sites. Protein crystal structure [PDB∶2JIU] shown as cartoon, with sites G724, T725, L858 and L861 shown as spheres. Structural regions highlighted in yellow are kinase subdomain I and the activation loop. The structure image was generated using PyMOL [75].

We looked at the features associated with each of the highly ranked mutations to understand how the combined classifier predicted these mutations to be cancer-associated (Table 5). The predicted mutation sites were visualized on a solved crystal structure of EGFR [PDB∶1JIU] using PyMOL [75] to view the structural context of each mutation (Figure 1).

**Table 5:** Feature values of selected mutations.

Mutation	E746K	L861R	L858Q	G724S	T725M
Protein Family	EGFR	EGFR	EGFR	EGFR	EGFR
Protein Group	TK	TK	TK	TK	TK
Wildtype amino acid	E	L	L	G	T
Blosum62	1	−2	−2	0	−1
Side Chain Polarity Mut	1	1	1	1	0
Conservation AllKinase Wild	0.049284	0.12363	0.409919	0.545306	0.122923
Conservation Group	0.392157	0.215686	0.803922	0.94902	0.380392
Conservation AllKinase	0.186021	0.070003	0.51031	0.716841	0.146852
Conservation Family	0.454545	0.818182	0.818182	1	0.909091
Sub domain	II	VIb	VIb	I	I
Avg Mass	146.18934	174.20274	146.14594	105.09344	149.20784
binding site	NA	NA	NA	NA	NA
Van der Waals Volume Wild	109	124	124	48	93
modification	NA	NA	NA	NA	Phosphorylation
snp amino acid	K	R	Q	S	M
Side Chain Polarity Wild	1	0	0	0	1
Is Pk Domain	0	0	0	0	0

The two top-scoring rare EGFR mutations are L861R and G724S. L861R (rank #1) occurs at the same site as another known driver, L861Q [76], in the activation segment of the kinase domain. L861Q is a frequently observed mutation in EGFR in lung cancer and this mutation is known to activate EGFR [77]; however, the functional impact of the rare L861R is not well understood.

G724S (rank #2) alters a conserved GxGxxG motif in the glycine-rich loop. However, it is the least conserved among the three glycines and does not directly participate in phosphoryl transfer [78], [79]. We note that there are other naturally occurring, active kinases which also have a serine at this position, such as 3-phosphoinositide dependent protein kinase-1 (PDPK1), calcium/calmodulin-dependent protein kinases (CAMK1a, CAMK2b), casein kinase II (CK2a, CK2b), mitogen-activated protein kinase kinase kinase 1 (MAP3K1), dual-specificity testis-specific protein kinase (TESK1, TESK2) and Tousled kinase 1 (TLK1) and 2 (TLK2).

In addition to the two high-scoring mutations we also chose additional mutations for experimental analysis (T725M, E746K and L858Q). Specifically, we chose mutations whose impact on EGFR structure and function was not obvious from crystal structure analysis.

T725M (rank #21) is another mutation in kinase subdomain I, in the *β*2 strand adjacent to the glycine-rich loop. From a structural perspective this prediction is surprising, given that the residue is solvent-exposed, oriented away from the kinase ATP-binding pocket and does not occur in the active site (Figure 1). The mutation type is not particularly drastic (BLOSUM62 score -1), and the conservation of this site is not strong outside across the protein kinase superfamily. It is unclear how mutation of this threonine to a methionine could impact kinase activity. However, the site is strongly conserved within the EGFR family (91%), and is a potential phosphorylation site [80], [81]. The trained classifiers appear to have used these features to assign a high probability of this mutation to be causative.

L858Q (rank #25) occurs in the kinase activation loop, like L861R (above), and is the site of another frequently observed lung cancer mutation, L858R [11].

E746K (rank #161) occurs in subdomain II but is spatially close to T725, C-terminal to the kinase-conserved VAIK motif, and also solvent-exposed in the EGFR structure (Figure 1). Although the ranking is low among other EGFR mutations, the priority score is 0.6178, indicating that it is predicted as more likely than not to be cancer-associated.

### T725M, E746K and L861R are activating EGFR mutations

**Figure 2:**
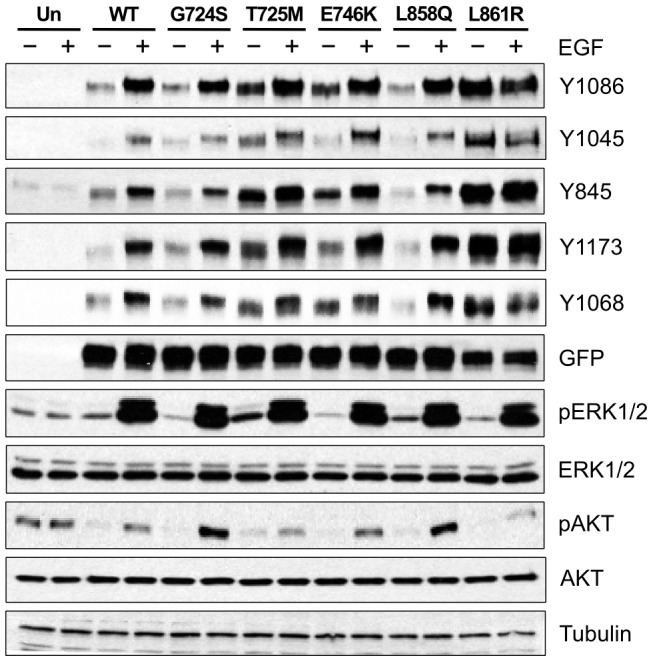
Auto-phosphorylation of wild-type and mutant EGFR and impact of mutations on downstream EGFR signaling. The blot shows phosphorylation of the four C-terminal tail tyrosines (Y1086, Y1045, Y845, Y1173 and Y1068) in EGFR, and two downstream proteins, ERK1/2 and AKT, in the presence (+) and absence(−) of EGF. “Un” indicates untransfected CHO cells. Total levels of EGFR (GFP), ERK1/2, AKT and tubulin (control) are also shown.

**Figure 3:**
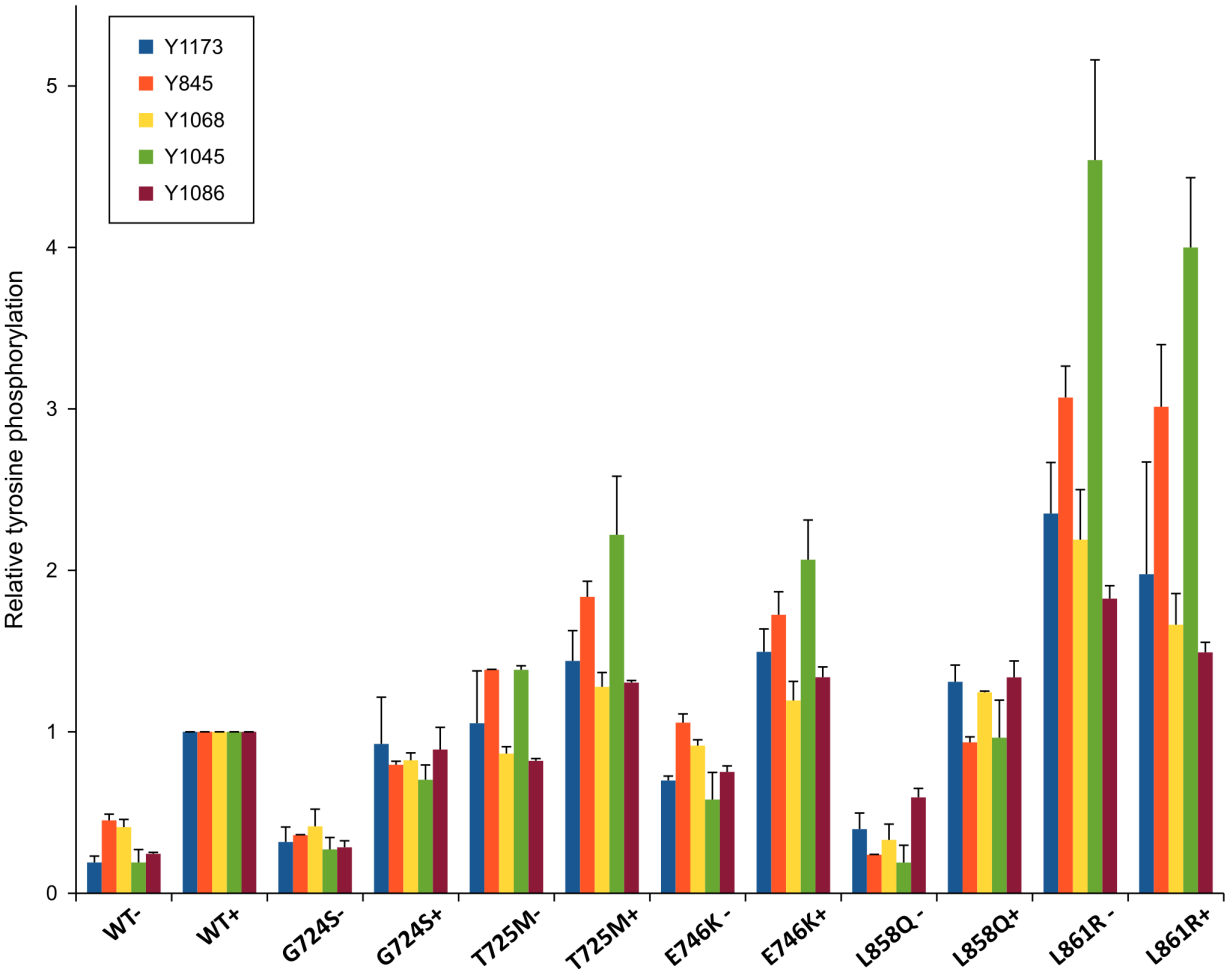
Quantified tyrosine auto-phosphorylation levels of wild-type and mutant-type EGFR. Quantified phosphorylation levels are shown in the form of histograms. Quantification was done using Image J.

EGFR point mutations T725M, E746K and L861R showed increased auto-phosphorylation compared to WT as shown in Figure 2. T725M and L861R resulted in hyper-phosphorylation at almost all the sites examined, i.e. Y1086, Y1045, Y845, Y1173 and Y1068. E746K, however, showed enhanced phosphorylation at Y1068, Y845, Y1173 and Y1068. The mutations G724S and L858Q, ranked 2 and 25 respectively (Table 4), did not show significant difference in C-terminal tail autophosphorylation compared to wild-type EGFR (Figure 2; Figure 3). However, G724S and L858Q showed elevated levels of AKT phosphorylation in the presence of EGF compared to WT and other mutants (Figure 2).

## Discussion

In this study, we reported novel features and a multiple classifier approach for identifying cancer-associated mutations in the cancer kinome. Our studies revealed that: (i) the depth of conservation of the mutated residue is a useful, novel feature for predicting cancer-associated mutations; (ii) combining multiple classifiers can improve prediction accuracies; and (iii) our novel features and multiple classifiers can be effectively applied in the identification of rare mutations in EGFR.

Mutational activation of EGFR is implicated in many cancers including lung, head and neck cancer, and clinical and cancer genome sequencing studies have identified hundreds of mutations in the protein kinase domain. However, much of the focus thus far has been on a handful of frequently observed mutations such as L858R and L861Q, while relatively little is known about the many rare mutations in EGFR such as T725M. The scoring scheme and multiple classifier approach we have introduced here help identify key rare mutations for follow-up experimental studies.

In particular, our studies suggest T725M as a likely cancer-associated mutation because it increases EGFR auto-phosphorylation activity in comparison to wild-type and other activating mutations such as L858R. The impact of the T725M mutation cannot be predicted by existing structural or functional information alone, and clinical samples do not currently highlight this as a highly recurrent mutation, as it appears only once in COSMIC v.50 and twice in COSMIC v.57. T725, however, is predicted to be a likely phosphorylation site [80], [81]. Thus, it is possible that the T725M mutation activates the kinase through loss of an inhibitory phosphorylation site. Indeed, mutational gain or loss of phosphorylation sites has been previously noted in cancer datasets [82], [83].

The frequently observed mutation L861Q is known to activate EGFR, but the impact of the rare L861R is not known. Here we showed that L861R activates EGFR in the absence of the activating EGF ligand, suggesting that it is also likely to be cancer-associated.

L858Q and G724S are two predicted mutants that do show appreciable change in EGFR autophosphorylation (Figure 2; Figure 3). This does not necessarily mean that these mutations are not causative, as these mutations can alter other aspects of EGFR signaling not considered in our studies. For example, recent studies showed that cancer mutations alter the temporal regulation and phosphorylation rates of the C-terminal tail tyrosines in EGFR [84]. Such changes in temporal regulation can contribute to abnormal downstream signaling without appreciable change in the level of C-terminal tail phosphorylation. Consistent with this view, the G724S and L858Q mutants increase phosphorylation of AKT despite little or no change in EGFR autophosphorylation (Figure 2). Although these observations must be further investigated through *in vitro* studies, the machine learning approach appears have used multiple correlative features to predict the causativeness of these mutants (see Table 5).

L858R is a recurrent lung cancer mutation which activates EGFR and also impacts drug binding [11]. This information perhaps contributed to the classification of L858Q as cancer-associated. However, our mutational experiments revealed that the L858Q mutation does not significantly alter the levels of EGFR autophosphorylation. However, as mentioned above, L858Q does alter downstream AKT phosphorylation (Figure 2). The context of L858Q suggests that the activation loop of EGFR is a frequent site of activating mutations; however, the L858Q mutation appears to alter downstream signaling in a manner distinct from L858R.

As our catalog of known drivers improves we can further improve our prediction system, using additional features such as protein dynamics and atomic details, and machine learning techniques such as semi-supervised learning [85] and clustering [86] to build a more sophisticated model to differentiate between causative and non-causative mutations in cancer. Moreover, our work could be extended to a prediction tool with clinical value, as well as provide a basis for further investigation into the relationship between protein evolution and disease.

## Supporting Information

Dataset S1 **Detailed experiment records and results.** Complete data sets, outputs and log files for all computational experiments. These files can be loaded in Weka [26] to reproduce the results presented in this paper.
(ZIP)

Table S1 **Descriptions of experiment designs and corresponding records.** Descriptions of the underlying data sets for each computational experiment, including an index for the raw data in Supplementary Dataset S1.
(XLSX)

Table S2 **Feature selection results.** The detailed feature selection results obtained from the 5 selection methods with 10-fold cross-validation.
(XLSX)

Table S3 **Ranking of 177 single-observation EGFR mutations in COSMIC v50.**
(CSV)

Table S4 **Ranking of 165 single-observation EGFR mutations in COSMIC v57.**
(CSV)

Table S5 **Ranking of the 71 single-observation EGFR mutations in COSMIC v50 that were observed more than once in COSMIC v57.**
(CSV)

Text S1 **Supplementary description of methods.** Detailed description of methods and alternative approaches.
(PDF)
