# Supplementary material for: Prediction and Prioritization of Rare Oncogenic Mutations in the Cancer Kinome Using Novel Features and Multiple Classifiers
Source: PLoS Comput Biol. 2014 Apr 17;10(4):e1003545. doi: 10.1371/journal.pcbi.1003545 (PMC3990476; doi:10.1371/journal.pcbi.1003545)
Supplement: Text S1 — Supplementary description of methods. Detailed description of methods and alternative approaches. (PDF) [file pcbi.1003545.s007.pdf]

Supplementary Information

ManChon U, Eric Talevich, Samiksha Katiyar, Khaled Rasheed, Natarajan Kannan

Accompanying the article: "Prediction and Prioritization of Rare Oncogenic Mutations in the Cancer Kinome Using Novel Features and Multiple Classifiers."

Contents

1 Data sources & definitions **2**

2 Feature selection **2**

2.1 Improvement on Performance with our Novel Features . . . 3

3 Experiment I: Evaluation of classifiers using COSMIC v.50 **5**

3.1 Cost-Sensitive Classifier . . . 6

3.2 Comparison of COSMIC All and COSMIC FG1 . . . 6

3.3 Ranking of 177 Putative EGFR Mutations - COSMIC v.50 . . . 7

3.3.1 Majority voting approach . . . 7

3.3.2 Weighted voting approach . . . 8

4 Experiment II: Comparison of COSMIC v.50 and v.57 **9**

4.1 Methods . . . 9

4.2 Results . . . 10

5 Experiment III: Evaluation of classifiers using COSMIC v.57 **11**

5.1 Removal of Cost-Sensitive Classifier . . . 13

5.2 Performance of individual classifiers with Experiment Setting III . . . 13

5.3 Performance of single and combined classifiers . . . 13

5.4 ROC curve and contribution of novel features . . . 16

5.5 Ranking of 165 Putative EGFR Mutations (COSMIC v.57) - Weighted Voting Approach 16

5.6 Alternative Ranking and Analyses of EGFR Mutations . . . 16

5.6.1 Ranking Single-Occurance Mutations in COSMIC v.50 Replicated in COSMIC v.57 . . . 17

5.6.2 Ranking with all COSMIC v.57 EGFR mutations witheld as a test set . . . 18

6 The Unsupervised Learning Module **19**
6.1 Learning Methods 19
6.1.1 Expectation-Maximization (EM) Algorithm 19
6.1.2 Methodology 20
6.2 Using Supervised and Unsupervised Learning Outputs to Identify Suspicious Mutations in COSMIC v.57 20
6.2.1 Identifying Suspicious Mutations in COSMIC-FG1 v.57 21

1 Data sources & definitions

The data sources for training and testing the classifiers are described in the main manuscript this supplementary text accompanies. Here, we describe the detailed methodology and results of several alternative computational experiments.

We use the following terms:

- **COSMIC-FG1**: The positive set of mutations in COSMIC that are observed in more than one distinct sample (**F**requency **G**reater than **1**). The corresponding negative set is the non-synonymous mutations obtained from SNP@Domain.
- **COSMIC-ALL**: The positive set consists of all nonsynonymous COSMIC mutations that appear in the protein kinase domain, i.e. omitting the filter applied in COSMIC FG1. The corresponding negative set is the non-synonymous mutations obtained from SNP@Domain.
- **COSMIC-FE1**: The "unconfirmed" set of mutations in COSMIC that are observed only once (**F**requency **E**qual to **1**).
- **Experiment I, II, III**: Designations for specific input training sets and filters used to train the classifiers and perform computational experiments.

Experiment settings, inputs and associated statistics are described for COSMIC v.50 in Tables III and V, and for COSMIC v.57 in Tables XI, XII and XIII. The settings and results used in the main text correspond to Experiment III.

2 Feature selection

We applied the following feature selection algorithms to evaluate our attributes:

- OneR algorithm [1], with a minimum bucket size of 14. This algorithm evaluates the contribution of an attribute by using the minimum-error attribute for prediction. It requires discretization if the values of an attribute are numeric. It generates a single level decision tree (a set of rules) which tests one particular attribute each time.

- Relief-based selection [2], with 10 nearest neighbors for attribute estimation. This algorithm is an instance-based algorithm which evaluates the worth of an attribute by repeatedly sampling N instances and considering the value of the N nearest instance of the same and different classes.

- Chi-Square selection. This algorithm evaluates the worth of an attribute by computing the value of the chi-squared statistic with respect to the class.

- Gain-ratio-based filter approach [3], using the Gain Ratio Attribute Evaluator and Spread Subsample method. Gain Ratio evaluates the worth of an attribute by measuring the gain ratio with respect to the class.

- Correlation-based selection [4], with Greedy (forward) searching algorithm. This approach performs a greedy forward search through the space of attribute subsets. Starting with no attributes in the space, and stoping when the addition of any remaining attributes results in a decrease in evaluation. The main idea of correlation-based selection algorithm is to find a feature subset which includes features that are highly correlated to the target attribute and have very low correlation to other features.

These methods were each applied separately as described in the main text on both the COSMIC-All and COSMIC-FG1 datasets. The results of feature selection on both these sets are shown side-by-side in Table I.

2.1 Improvement on Performance with our Novel Features

Based on the feature selection results, we additionally removed our proposed novel features in order to evaluate their impact on prediction (see Section 5.4):

- Conservation_Consensus_AllKinase
- Conservation_Wild_AllKinase
- Conservation_Consensus_Family
- Conservation_Consensus_Group
- Protein_Family
- Protein_Group

Table I. Selected features based on v.57 COSMIC All and FG1 datasets.
The "Votes" column indicates how many feature selection algorithms cast a vote for that particular feature during the 10-fold cross-validation selecting procedure; the "Avg Rank" column describes the averaged rank of a particular feature within the selected algorithms. The feature "binding site" was selected with COSMIC-All but not COSMIC-FG1, and "Isoelectric point, WT" was selected with COSMIC-FG1 but not COSMIC-All.

Feature	Votes		Avg Rank	
	All	FG1	All	FG1
Protein kinase family	5	5	1.40	1.20
Protein kinase group	5	4	1.80	2.25
Amino acid type, WT	5	5	8.00	7.80
BLOSUM62 pairwise score	5	4	8.20	8.25
Side-chain polarity, mutant	5	3	11.00	11.67
Conservation of wild type in all kinases	5	4	11.60	6.00
Conservation of consensus type in kinase group	5	4	11.60	10.50
Conservation of consensus type in all kinases	5	4	13.00	13.00
Conservation of consensus type in kinase family	4	5	5.75	4.60
Kinase subdomain	4	5	6.00	3.60
Average mass of amino acid, WT	4	5	7.50	7.80
Is a binding site?	4	—	8.25	—
Van der Waals volume, WT	4	5	8.75	9.80
Site modification type (if any)	4	3	9.25	10.67
Amino acid type, mutant	4	4	10.75	10.00
Side-chain polarity, WT	4	4	11.50	13.25
Is in protein kinase domain?	3	4	11.67	16.25
Isoelectric point, WT	—	4	—	11.00

Table II. Comparison of performance of individual and combined classifiers on COSMIC-FG1 v.57 with our proposed novel features removed

Algorithms	TP Rate	FP Rate	Accuracy	Precision	Recall	F-Measure
J48 (Tree)	0.646	0.187	0.745	0.702	0.646	0.673
Random Forest	0.650	0.236	0.754	0.653	0.650	0.652
NB Tree	0.637	0.202	0.740	0.682	0.637	0.659
Functional Tree	0.646	0.263	0.727	0.627	0.646	0.636
Decision Table	0.664	0.202	0.758	0.691	0.664	0.677
DTNB	0.655	0.218	0.803	0.673	0.655	0.664
LWL(J48+KNN)	0.699	0.215	0.754	0.690	0.699	0.695
Bayes Net	0.584	0.202	0.725	0.663	0.584	0.621
Naive Bayes	0.597	0.227	0.688	0.643	0.597	0.619
SVM	0.655	0.218	0.729	0.673	0.655	0.664
Neural Network	0.619	0.245	0.749	0.633	0.619	0.626
Combined (0.5)	0.876	0.094	0.894	0.865	0.876	0.870

3 Experiment I: Evaluation of classifiers using COSMIC v.50

The settings for Experiment I are described in Table III. Table IV shows the accuracy of the 11 different classifiers trained and tested on the two positive training datasets (COSMIC-FG1 and COSMIC-All, see below) for models that were trained with all features and those trained with only selected features.

Table III. Experiment setting I.
Based on COSMIC v.50 dataset.

Exp. #	Training Set	Testing Set (Prediction)
I.1	Mutations (Kinase domain) in COSMIC v.50 dataset, excludes 177 EGFR mutations in the COSMIC v.50 dataset whose frequency is equal to 1	177 EGFR mutations in the COSMIC v.50 dataset whose frequency is equal to 1
I.2	Mutations (Kinase domain) in COSMIC v.50 dataset whose frequency is greater than 1	177 EGFR mutations in the COSMIC v.50 dataset whose frequency is equal to 1

In general, 10 out of 11 classifiers were more accurate when trained only with the selected features, with an average 0.59% improvement in experiment I.1 and 0.55% improvement in Experiment I.2. These results support the effectiveness of our selected features. In addition, the differences in accuracy between the 11 classifiers are very subtle within each dataset, though DTNB and SVM perform slightly better than others in Experiment I.1, while SVM and Functional Tree performs slightly better than others in Experiment I.2.

Table IV. Accuracy of 11 trained models - Experiment I.

Algorithm	All Features		Selected Features	
	I.1	I.2	I.1	I.2
J48 (Tree)	84.4142	96.2312	84.6234	96.2312
Random Forest	83.1590	94.7236	84.6234	96.4824
NB Tree	82.3222	93.2161	84.1004	93.4673
Functional Tree	83.5774	96.4824	84.8326	96.4824
Decision Table	86.1925	88.1910	86.1925	88.1910
DTNB	87.5523	94.4724	87.5523	93.9698
LWL(J48+KNN)	83.5774	94.4724	85.1464	95.4774
Bayes Net	84.8326	95.7286	84.9372	95.7286
Naive Bayes	83.5774	92.4623	83.5774	94.9749
SVM	87.3431	96.7337	87.1339	96.7337
Neural Network	83.4728	94.9749	83.7866	95.9799

3.1 Cost-Sensitive Classifier

In the experimental design using COSMIC FG1 (Exp. I.2), the positive and negative training sets are highly imbalanced: the number of instances in the non-disease set is almost 5 times more than the disease set (67 vs. 331; see Table V). Since highly imbalanced datasets can lead to inferior classification results [5], it is critical to compensate for such an imbalance before performing any classification.

Table V. Number of training instances and corresponding splits.
For Experiment Setting I. Based on the COSMIC v.50 dataset.

Condition	Disease	Non-disease	Total
COSMIC All (Exp. I.1)	625	331	956
COSMIC FG1 (Exp. I.2)	67	331	398

Thus, for this experiment, on top of each classifier that we utilized, we applied the Cost-Sensitive Classifier for defining the cost matrix according to the imbalance ratio of the dataset. We defined the False Negative cost in the cost matrix as the ratio between the majority class (in this case, the negative set, non-causative mutations) and the minority class (the positive set, causative mutations).

3.2 Comparison of COSMIC All and COSMIC FG1

Table VI presents the experimental results in terms of confusion matrix and several other measurement indexes which quantify the performance of the individual classifiers.

The performance of all these classifiers is lower on COSMIC All than on COSMIC FG1 dataset. One possible explanation for this is that COSMIC-All may contain some passengers in the positive set, blurring the distinction between the positive and negative sets during training.

On another note, although the accuracies of the models trained with the COSMIC FG1 dataset are about 10% higher than those with the COSMIC-All dataset, this result should be taken with some

Table VI. Confusion matrix.
Classifiers trained with selected features on the COSMIC-All dataset.

Algorithms	TP	FN	TN	FP
J48 (Tree)	557	68	252	79
Random Forest	568	57	241	90
NB Tree	550	75	254	77
Functional Tree	562	63	249	82
Decision Table	588	37	236	95
DTNB	577	48	260	71
LWL(J48+KNN)	555	70	259	72
Bayes Net	544	81	268	63
Naive Bayes	539	86	260	71
SVM	566	59	267	64
Neural Network	573	52	228	103

caution because the dataset COSMIC FG1 is much smaller than COSMIC-All, and therefore the trained models of COSMIC FG1 may be less generalizable than those of COSMIC-All. We therefore considered the possibility that the trained models in COSMIC FG1 might not outperform the models of COSMIC-All when applied to new, unseen data. The procedure of 10-fold cross-validation is intended to address this concern by repeatedly reserving an "unseen" test dataset during the evaluation process. We also evaluated our assumption on the most recent COSMIC v.57 dataset, which includes mutations that were not available in COSMIC v.50 (see Section Alternative Ranking and Analyses of EGFR Mutations).

3.3 Ranking of 177 Putative EGFR Mutations - COSMIC v.50

3.3.1 Majority voting approach

According to this ranking, 100% (177/177) of the single-observed instance non-synonymous point mutations in the kinase domain of EGFR are likely drivers. Specifically, 74 mutations received "driver" votes from all 11 classifiers, 31 received 10 votes, 20 received 9 votes, 25 received 8 votes, 18 received 7 votes, and 9 received 6 votes - by a majority criterion, these would be considered likely drivers. Detailed distribution of the mutations is shown in Table VII and Figure I.

Table VII. Distribution of predictions for 177 EGFR mutations in COSMIC v.50 - majority voting approach

Number of Votes	Number of Mutations	Predicted Class
11	74	Driver
10	31	
9	20	
8	25	
7	18	
6	9	

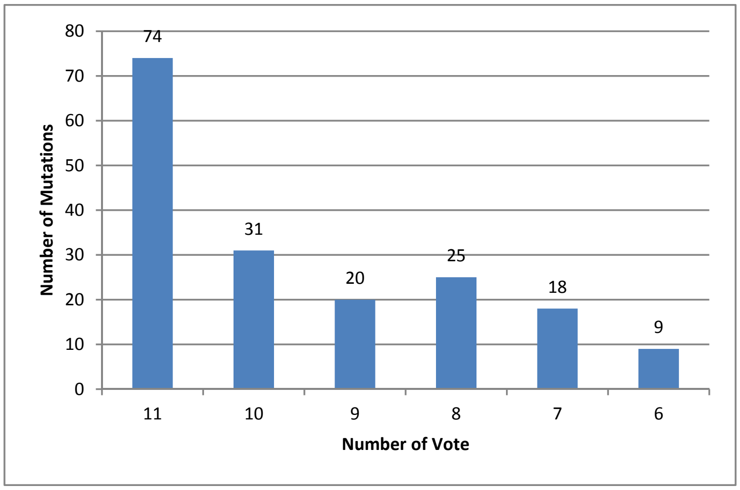

Figure I. Distribution of predictions for 177 EGFR mutations in COSMIC v.50.
Mutations were prioritized with the combined classifiers trained with Experiment Setting I and scored using the majority voting approach.

3.3.2 Weighted voting approach

Based on this improved ranking approach, if we posit 50% as a threshold to differentiate "Driver" and "Passenger", 175 mutations were given more than 50% probability to be "driver" from all 11 classifiers while other 2 with less than 50%. Specifically, 64 mutations ranked between 90% and 100%, 41 ranked 80% to 89.99%, 27 ranked 70% to 79.99%, 31 ranked 60% to 69.99%, 12 ranked 50% to 59.99% - by a applying 50% as threshold, these would be considered likely drivers. Of the other mutations, which are likely passengers, 2 ranked between 40% and 49.99%. It is clear that the weighted approach gave us a more informative prediction result, in terms of indicating the possible probability of each mutation that it is likely to be a driver or a passenger. Detailed distribution of the ranked mutations is illustrated in Table VIII. and corresponding visualization is given at Figure II. The complete list of ranking of these 177 putative EGFR mutations is given in Supplementary Table S26.

Table VIII. Distribution of predictions for 177 EGFR mutations in COSMIC v.50 - weighted voting approach.

Probability (%)	Number of Mutations	Predicted Class
90 - 100	64	
80 - 89.99	41	
70 - 79.99	27	Driver
60 - 69.99	31	
50 - 59.99	12	
40 - 49.99	2	Passenger

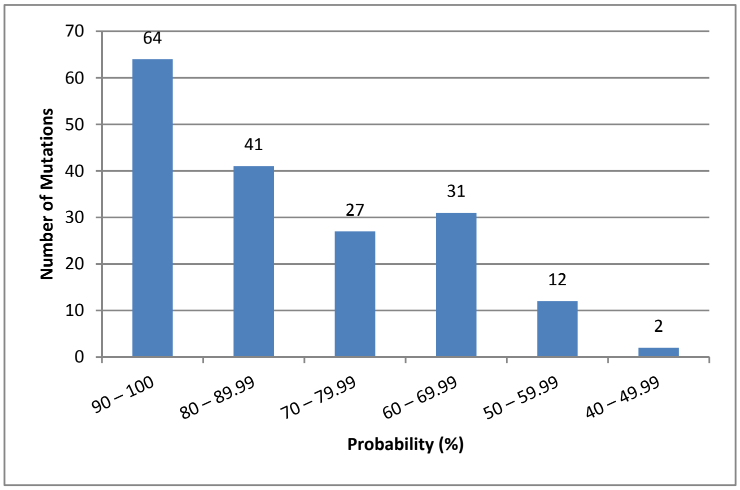

Figure II. Distribution of predictions for 177 EGFR mutations in COSMIC v.50 Dataset. Mutations were scored using the weighted voting approach.

4 Experiment II: Comparison of COSMIC v.50 and v.57

After our inital analysis with COSMIC version 50, we updated our analyses with COSMIC version 57. We then wondered how well our previously trained model (with COSMIC v.50 FG1 as the positive set) predicted the 71 EGFR mutations that appear only once in COSMIC v.50 but appear more than once in COSMIC v.57 (Table IX - Experiment Setting II), as an additional test of the validity of our use of COSMIC-FG1 as a positive set for training the predictive models.

Table IX. Experiment Setting II.
Based on COSMIC v.57 dataset.

Exp. #	Training Set	Testing Set (Prediction)
II.1	Mutations (Kinase domain) in COSMIC v.50 dataset whose frequency is greater than 1	71 EGFR mutations that appear only once in COSMIC v.50 but more than once in COSMIC v.57

4.1 Methods

In order to provide more evidence to support the rationale on our selection of the models trained on the well-performing positive sets (COSMIC mutations observed in more than one sample, as described in the main text) for EGFR prioritization, we present a more detailed analysis in this section with the updated COSMIC v.57 dataset.

The statistics of the new dataset are presented in Table XIII. Between COSMIC versions 50 and 57, the number of “disease” instances has increased in the COSMIC-All table by nearly double and

the in COSMIC-FG1 table by threefold. As the dataset based on COSMIC v.57 is relatively well balanced between positive and negative instances for training, the Cost-Sensitive Classifier is not needed here.

In the COSMIC dataset version 50 (v.50), there are 177 EGFR mutations that were observed in only one unique sample, while the most updated COSMIC dataset (v.57) includes 165 such single-observation mutations; 106 of these mutations are the same among these two versions of the COSMIC dataset. Furthermore, 71 EGFR mutations that appear only once in COSMIC v.50 appear more than once in COSMIC v.57, and there are 59 new EGFR mutations that appear only once in v.57 and did not appear in v.50.

4.2 Results

The result of the comparison is presented in Table X and its corresponding visualization is illustrated in Figure III. Using our previously trained model, of the 71 EGFR mutations whose frequency is equal to 1 in COSMIC v.50 but greater than 1 in COSMIC v.57, 65 were predicted as causative and the remaining 6 were predicted non-causative. This result further supports our assumption that mutations appear more than once in the COSMIC dataset are more likely to be causative than those appear only once. The complete list of these 71 mutations is given in Supplementary Table S28.

Table X. Distribution of predictions for 71 EGFR mutations observed once in COSMIC v.50 but more than once in COSMIC v.57 - weighted voting approach.
Predictions are the probability that a mutation is cancer-associated. Mutations with probability above 50% are considered likely cancer-associated ("driver," as opposed to "passenger").

Probability (%)	Number of Mutations	Predicted Class
90 - 100	29	Driver
80 - 89.99	18	
70 - 79.99	6	
60 - 69.99	9	
50 - 59.99	7	
40 - 49.99	2	Passenger

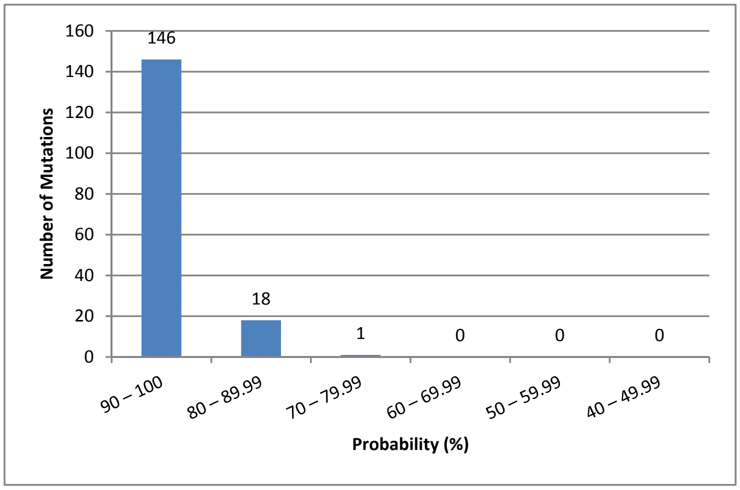

Figure III. Prediction probabilities of 71 EGFR mutations observed once in COSMIC v.50 but more than once in COSMIC v.57.

5 Experiment III: Evaluation of classifiers using COSMIC v.57

Based on the various experiments presented in previous sections, we designed more *in silico* experiments with different combination of training and testing datasets to thoroughly analysis the robustness our combined multiple classifiers method, as well as to provide clearer guidance for the usage of our prediction results for further analysis. All experiments present in this section is based on the most updated COSMIC dataset, version 57. The detailed settings of the experiments are summarized in Table XI, and the statistics of the dataset for experiment III are presented in Table XII.

The positive dataset for training in experiment III.1 includes all non-synonymous mutations (occuring in the kinase domain) in COSMIC v.57, by excluding 165 EGFR mutations whose frequency is equal to 1 for testing. Experiment III.2 and III.3 are designed to use the mutations (Kinase domain) in COSMIC v.57 dataset whose frequency is greater than 1 as positive set, to predict 165 EGFR mutations in the COSMIC v.57 dataset whose frequency is equal to 1, and the 106 EGFR mutations that commonly appears in both the COSMIC v.50 and v.57 dataset with frequency equal to 1 respectively. Experiment III.1 and IIII.2 are the updates of Experiment I.1 and I.2 with the new version dataset. Experiments III.3 is a subset of III.2 which provides the reader a clear view of the ranking of the mutations whose frequencies have not changed between COSMIC v.50 and v.57.

The positive dataset for training of experiment III.4, III.5, and III.6 includes mutations (Kinase domain) in COSMIC v.57 dataset whose frequency is greater than 1, by excluding the 71 EGFR mutations whose frequency was 1 in COSMIC v.50 but later on increased to more than 1 in the v.57 dataset. Experiment III.4 is very interesting because testing dataset shows how those 71 EGFR

Table XI. Experiment Setting III - based on COSMIC v.57.
All training sets derived from COSMIC v.57, restricted to point mutations occurring in the kinase domain.

Experiment	Training Set	Testing Set (Prediction)
III.1	Exclude 165 EGFR mutations in the COSMIC v.57 dataset whose frequency is equal to 1	165 EGFR mutations in the COSMIC v.57 dataset whose frequency equal to 1
III.2	Frequency greater than 1	165 EGFR mutations in the COSMIC v.57 dataset whose frequency is equal to 1
III.3	Frequency greater than 1	106 EGFR mutations that appears in both the COSMIC v.50 and v.57 dataset whose frequency is equal to 1
III.4	Frequency greater than 1, exclude the 71 EGFR mutations whose freq were 1 in v.50 but > 1 in v.57	71 EGFR mutations that appear only once in COSMIC v.50 turn into appear more than once in COSMIC v.57
III.5	Frequency greater than 1, exclude the 71 EGFR mutations whose freq were 1 in v.50 but > 1 in v.57	177 EGFR mutations in the COSMIC v.50 dataset whose frequency is equal to 1
III.6	Frequency greater than 1, exclude the 71 EGFR mutations whose freq were 1 in v.50 but > 1 in v.57	165 EGFR mutations in the COSMIC v.57 dataset whose frequency is equal to 1
III.7	Frequency greater than 1, exclude all EGFR mutations	165 EGFR mutations in the COSMIC v.57 dataset whose frequency is equal to 1
III.8	Frequency greater than 1, exclude all EGFR mutations	All (253) EGFR mutations in the COSMIC v.57 dataset (includes those in COSMIC v.50)

Table XII. Number of training instances and corresponding splits of Experiment Setting III - COSMIC v.57.
Training set sizes obtained from COSMIC v.57 and SNP@Domain under several criteria.

Experiment	Disease	Non-disease	Total
III.1	1084	331	1415
III.2 / 3	226	331	557
III.4 / 5 / 6	155	331	486
III. 7 / 8	138	326	464

mutations that appear only once in COSMIC v.50 turn into appear more than once in COSMIC v.57 are classified. This is also a further confirmation to Experiment II.1, in where we use COSMIC v.50 FG1 as training dataset. Experiment III.7 and III.8 should be given some attention as they help to clear the suspicion of the training set of our previous experiments. The training set of previous experiments include EGFR mutations whose frequency is greater than one, while all the EGFR mutations are completely excluded from the training set in these experiments, and the testing set (prediction) includes 165 EGFR mutations whose frequency is equal to one (III.7) and all EGFR mutations regardless of their frequencies (III.8). In sum, III.7 is a subset of III.8.

5.1 Removal of Cost-Sensitive Classifier

The COSMIC v.57 update increased the sized of the positive set, reducing the imbalance relative to the size of the negative set (226 vs. 331, see Table XIII). As this imbalance is less severe, the Cost-Sensitive Classifier was not needed for the *in silico* experiments based on that dataset.

Table XIII. Number of training instances and corresponding splits.
For Experiment Setting II, based on COSMIC v.57 dataset.

Condition	Disease	Non-disease	Total
COSMIC-All	1249	331	1580
COSMIC FG1	226	331	557

5.2 Performance of individual classifiers with Experiment Setting III

Table XIV shows the accuracy of the 11 trained classifiers with 10-fold cross-validation of Experiments III. Similarly, the differences of performance between the 11 classifiers are inconspicuous within each experiment, though SVM and tree classifiers perform slightly better in most cases.

Table XIV. Accuracy of 11 trained models with Experiment Setting III.

Algorithm	Experiment III #			
	1	2, 3	4, 5, 6	7, 8
J48 (Tree)	0.864	0.968	0.971	0.981
Random Forest	0.865	0.962	0.963	0.952
NB Tree	0.858	0.948	0.949	0.939
Functional Tree	0.852	0.969	0.969	0.978
Decision Table	0.880	0.930	0.919	0.918
DTNB	0.865	0.969	0.967	0.978
LWL(J48+KNN)	0.870	0.962	0.965	0.978
Bayes Net	0.840	0.959	0.959	0.974
Naive Bayes	0.834	0.946	0.947	0.946
SVM	0.878	0.973	0.971	0.976
Neural Network	0.830	0.968	0.959	0.961

5.3 Performance of single and combined classifiers

Table XV presents an analysis of the performance of the combined classifier using Experiment Setting III.2, setting the probability threshold for labeling a mutation as causative at incremental values. In the main text, the threshold 0.5 was chosen.

Table XVI presents the equivalent statistics for the classifiers trained under Experiment III settings (COSMIC v.57) without removing single-observation mutations, i.e. COSMIC-All.

Comparing the averaged F-measure of the training models between experiment III.1 and experiment I.1 (Table XVII), as well as experiment III.2 and experiment I.2 (Table XVIII), there are on average 0.51% and 0.9% improvement respectively. The underlying rationale of these

Table XV. Performance of combined classifier - COSMIC FG1 dataset with selected features.

Threshold	TP Rate	FP Rate	Precision	Recall	F-Measure
0.05	1	0.236	0.743	1	0.853
0.1	1	0.106	0.866	1	0.928
0.15	1	0.063	0.915	1	0.956
0.2	1	0.036	0.950	1	0.974
0.25	1	0.036	0.950	1	0.974
0.3	0.996	0.027	0.962	0.996	0.978
0.35	0.987	0.021	0.970	0.987	0.978
0.4	0.987	0.018	0.974	0.987	0.980
0.45	0.987	0.015	0.978	0.987	0.982
0.5	0.987	0.009	0.987	0.987	0.987
0.55	0.982	0.009	0.987	0.982	0.984
0.6	0.982	0.009	0.987	0.982	0.984
0.65	0.982	0.009	0.987	0.982	0.984
0.7	0.982	0.006	0.991	0.982	0.987
0.75	0.978	0.006	0.991	0.978	0.984
0.8	0.973	0.006	0.991	0.973	0.982
0.85	0.973	0.006	0.991	0.973	0.982
0.9	0.960	0	1	0.960	0.980

Table XVI. Performance of combined classifier - COSMIC-All dataset with selected features.

Threshold	TP Rate	FP Rate	Precision	Recall	F-Measure
0.05	1	0.601	0.845	1	0.916
0.1	1	0.526	0.862	1	0.926
0.15	1	0.435	0.883	1	0.938
0.2	0.999	0.402	0.891	0.999	0.942
0.25	0.998	0.363	0.900	0.998	0.947
0.3	0.997	0.290	0.918	0.997	0.956
0.35	0.993	0.257	0.927	0.993	0.959
0.4	0.988	0.218	0.937	0.988	0.962
0.45	0.984	0.190	0.944	0.984	0.964
0.5	0.978	0.163	0.952	0.978	0.965
0.55	0.967	0.139	0.958	0.967	0.962
0.6	0.958	0.112	0.966	0.958	0.962
0.65	0.940	0.060	0.981	0.940	0.960
0.7	0.919	0.039	0.987	0.919	0.952
0.75	0.893	0.021	0.993	0.893	0.940
0.8	0.857	0.012	0.996	0.857	0.921
0.85	0.797	0.003	0.999	0.797	0.887
0.9	0.724	0.003	0.999	0.724	0.840

Table XVII. Detailed measurement - COSMIC-All dataset with selected features.

Algorithms	TP Rate	FP Rate	Precision	Recall	F-Measure
J48 (Tree)	0.934	0.366	0.893	0.934	0.913
Random Forest	0.946	0.399	0.886	0.946	0.915
NB Tree	0.933	0.387	0.888	0.933	0.910
Functional Tree	0.903	0.317	0.903	0.903	0.903
Decision Table	0.957	0.372	0.894	0.957	0.924
DTNB	0.935	0.366	0.893	0.935	0.914
LWL(J48+KNN)	0.946	0.378	0.891	0.946	0.918
Bayes Net	0.877	0.284	0.910	0.877	0.893
Naive Bayes	0.875	0.302	0.905	0.875	0.890
SVM	0.943	0.332	0.903	0.943	0.922
Neural Network	0.892	0.372	0.887	0.892	0.890

improvements is due to the increment in the size of our dataset and the balance rate between the number of instances of the disease set and non-disease set, as well as how we select our training data (COSMIC-FG1). In the very beginning, we made the assumption of that mutations appear more than once in the COSMIC dataset are having higher possibility to be driver than those appear only once, and then we proved the correctness (in computational point of view) of this assumption by tracking how those mutations that appear only once in COSMIC v.50 but more than once in COSMIC v.57. Therefore, by comparing experiment III.1 to I.1 and experiment III.2 to I.2, it provides further evidence on why the balance dataset is important, as well as the rationale of selecting COSMIC-FG1 as training dataset could better improve the capability of generalization of our training models.

Table XVIII. Detailed measurement - classifiers trained with selected features on COSMIC-FG1 v.57 dataset.

Algorithms	TP Rate	FP Rate	Precision	Recall	F-Measure
J48 (Tree)	0.978	0.039	0.944	0.978	0.961
Random Forest	0.956	0.033	0.952	0.956	0.954
NB Tree	0.960	0.060	0.916	0.960	0.937
Functional Tree	0.960	0.024	0.964	0.960	0.962
Decision Table	0.982	0.106	0.864	0.982	0.919
DTNB	0.969	0.030	0.956	0.969	0.963
LWL(J48+KNN)	0.973	0.045	0.936	0.973	0.954
Bayes Net	0.978	0.054	0.925	0.978	0.951
Naive Bayes	0.965	0.066	0.908	0.965	0.936
SVM	0.969	0.024	0.965	0.969	0.967
Neural Network	0.965	0.030	0.956	0.965	0.960

5.4 ROC curve and contribution of novel features

The Receiver Operating Characteristic (ROC) curve illustrating the tradeoff of true positive and false positive rates, with and without the 6 novel, kinase-specific features introduced in this study, is shown in Figure IV.

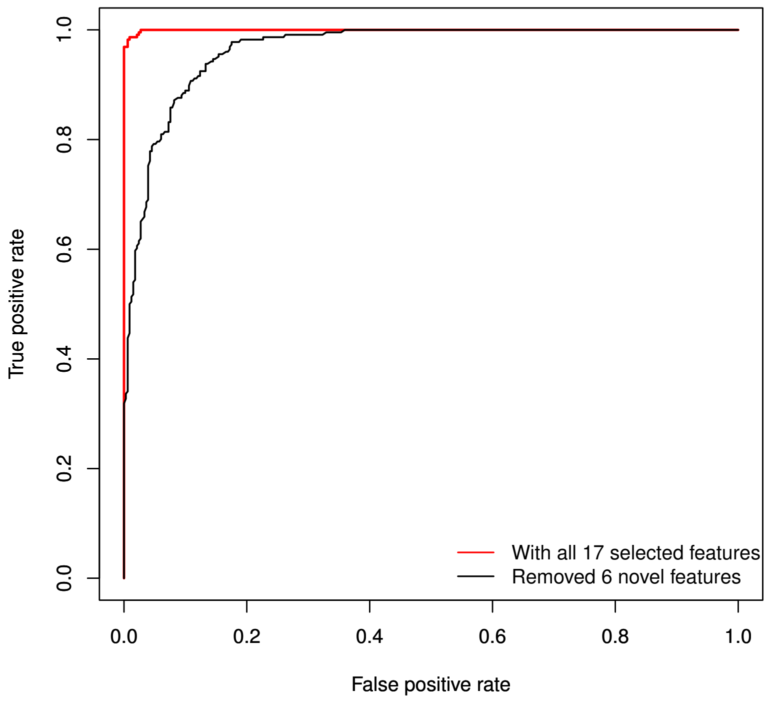

Figure IV. ROC Curve of the combined classifier with threshold 0.5 in COSMIC-FG1 v.57. Performance of the combined classifier is shown in red. Another version of the classifier trained without the 6 novel features introduced in this study is shown in black. The comparison of the two curves shows that the 6 features improve the performance of the classifier across the range of specificities and sensitivities.

5.5 Ranking of 165 Putative EGFR Mutations (COSMIC v.57) - Weighted Voting Approach

By applying experiment setting III.2 to the updated COSMIC v.57 dataset, the weighted voting approach predict all 165 single-observed instance non-synonymous point mutations in the kinase domain of EGFR are likely drivers. Specifically, 146 mutations ranked between 90% and 100%, 18 ranked 80% to 89.99%, 1 ranked 70% to 79.99%. Detailed distribution of the ranked mutations is illustrated in Table XIX and corresponding visualization is given at Figure V. The complete list of ranking of these 165 putative EGFR mutations is given in Supplementary Table S27.

5.6 Alternative Ranking and Analyses of EGFR Mutations

The experiments presented in this section use several different combinations of training and testing datasets to thoroughly analyze the robustness our combined multiple classifiers method, as well

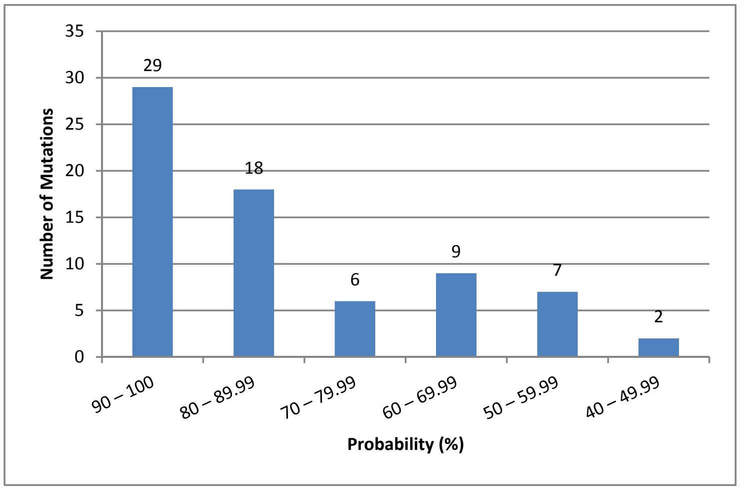

Figure V. Distribution of predictions for 165 EGFR mutations in COSMIC v.57 dataset.
Weighted voting approach.

Table XIX. Distribution of predictions for 165 EGFR mutations in COSMIC v.57 - weighted voting approach.

Probability (%)	Number of Mutations	Predicted Class
90 - 100	146	
80 - 89.99	18	
70 - 79.99	1	Driver
60 - 69.99	0	
50 - 59.99	0	
40 - 49.99	0	Passenger

as to provide clearer guidance to the reader for the usage of our prediction results in further analyses. These experiments are based on the most updated COSMIC dataset, version 57 (with some experiments referencing COSMIC version 50 for the purpose of filtering mutations).

The detailed records of experiments introduced in this section are given in Supplementary Dataset S1.

5.6.1 Ranking Single-Occurance Mutations in COSMIC v.50 Replicated in COSMIC v.57

Experiment III.4 is another interesting experiment which re-ranks the four mutations we picked in the previous section with the most updated training dataset. Other than mutation T725M, all other three previously selected mutations are still ranked highly by the classifiers that trained with the updated dataset (Table XX). However, T275M is assigned the rank probability of 81.7%, which still indicates a relatively high probability of being a driver mutation. A possible explanation of

the drop of its ranking is that, since the number of instances of the positive training set has been increased, more information of the mutations becomes available to the classifiers. Thus, while the classifiers found strong support for T275M as a cancer-associated mutation, still stronger support was found for the other mutations which are now ranked higher. Out of the top ranked 35 (top 50%) mutations in experiment II.1, 27 are still ranked within the top 35 in experiment III.4, meaning that the majority (77%) of the top-ranked unseen mutations did not change their ranking dramatically even though the training datasets are different.

Table XX. Probability score of the 4 selected mutations with Experiment Setting III.4.

Mutation	Probability	Rank
G724S	0.9295	1
T725M	0.8170	43
L858Q	0.9085	6
L861R	0.9137	5

5.6.2 Ranking with all COSMIC v.57 EGFR mutations witheld as a test set

In experiment III.7 we asked if all mutations of a single gene (EGFR in this case) are withheld from the training set, then how would the classifiers trained on mutations in other genes classify the mutations in the missing gene. The testing set of experiment III.7 is the 165 EGFR mutations in the COSMIC v.57 dataset whose frequency is equal to 1, therefore it is intuitive that we should select experiment III.2 for comparison as we have already proved the robustness of the training models in III.2. In the 165 single-observation EGFR mutations, we gradually selected the top 50% to 5% (with step size of 5%) ranked mutations from the predictions of experiment III.2 and III.7, then count the overlaps between them.

As illustrated in Figure VI, excluding all mutations that belong to a single gene from the training dataset does not result in a robust classifier. Ideally, the predictions of these two experiments should match fairly closely, but here we see the closest matching occurs at the top 50% threshold, at which point less than 80% of the ranked mutations between two prediction sets are match, and the proportion of matched predictions even drops to 12.5% at the top 5% threshold.

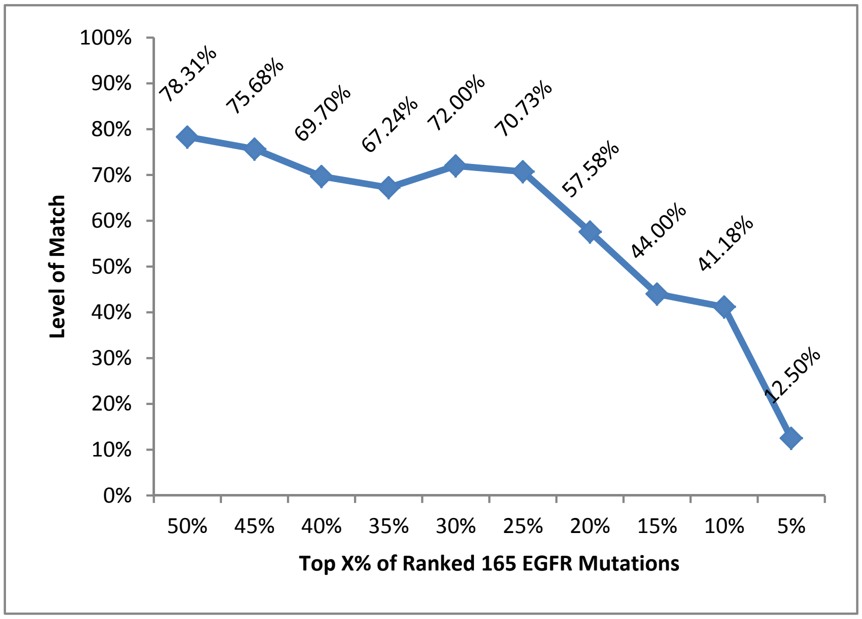

Figure VI. Level of match between Experiment III.2 and III.7.

6 The Unsupervised Learning Module

In this appendix, we first introduce the unsupervised learning methods used. We then present the use of the unsupervised module together with the supervised combined classifier to identify suspicious labeled instances in the COSMIC-FG1 table.

6.1 Learning Methods

In order to add another level of confidence about the relationship between different mutations in our dataset, thereby reducing the label uncertainty, we first use the Expectation-Maximization (EM) algorithm to cluster the mutations, followed by using our self-invented score to measure the level of oncogenicity of the mutations.

6.1.1 Expectation-Maximization (EM) Algorithm

Expectation-Maximization (EM) [6, 7] is a popular algorithm in data mining. It is an iterative method for finding maximum likelihood or maximum a posteriori (MAP) estimates of parameters in statistical models, where the model depends on unobserved latent variables. In short, EM finds clusters by determining a mixture of Gaussians to fit a given dataset.

The EM [8] algorithm works in two alternating steps: The expectation (E) step computes the expectation of the loglikelihood evaluated using the current estimate for the parameters; it calculates

the probability that each datum is a member of each cluster. The maximization (M) step computes parameters maximizing the expected log-likelihood found in the E step; it alters the parameters of each cluster to maximize those probabilities. These parameter-estimates are then used to determine the distribution of the latent variables in the next E step. The E step and the M step work iteratively until convergence, but there is no guarantee for perfect correctness.

6.1.2 Methodology

We first represent the labeled (COSMIC-FG1 v.57) and unlabeled (COSMIC-FE1) datasets by the 17 features selected using the feature selection methods described in the main text. We then randomize the labeled dataset, followed by randomly splitting the labeled dataset into 90% and 10%. We then combine the 90% split labeled data with the unlabeled dataset, apply the EM algorithm to this combined dataset to perform clustering. Afterwards, we use the trained EM model to cluster the split 10% labeled data. With the clustering outputs, we integrate the unlabeled dataset, and the labeled dataset (both the 90% and 10%) together for validity analysis. For each instance in this combined dataset, we first find the cluster it belongs to, and then we compute a score for it. We repeat the above steps for 10 times, and then we generate the averaged score for every single instance in both the labeled and unlabeled dataset, namely the "U-Score" (short for Unsupervised Score).

The U-score is a number between 0 and 1 which is proportional to how positive (i.e. causative) a mutation appears to be, based on clustering. The U-Score depends on two factors: 1) the proportion of positive instances in the cluster to which the mutation belongs; and 2) the proximity of the mutation to other positive and negative mutations in the same cluster. Thus, if a mutation falls in a cluster that has a majority of positive instances, and is closer to positive than negative instances will receive a high U-Score. We are currently in the process of preparing a manuscript about unsupervised learning applied to this domain, which will include more details.

6.2 Using Supervised and Unsupervised Learning Outputs to Identify Suspicious Mutations in COSMIC v.57

There are two scores in our system — namely the S-Score and the U-Score — that are generated by the supervised and the unsupervised learning modules respectively to measure the oncogenicity of a mutation. Both scores are scaled within 0 to 1, where a higher score stands for a higher probability for the corresponding mutation to be causative. The S-Score is a probability score generated through combining 11 well established classifiers, with 10-fold cross-validation accuracy as weight as described in the main text. Based on the clustering results generated in the unsupervised learning module, the U-Score is a number which measures the similarity of a mutation to the causative and non-causative mutations in the same cluster it belongs to.

Since there exists a certain level of uncertainty in the labels ("Causative" and "Non-Causative") of our dataset, the predictive model that is trained by the supervised learning module might be biased. Therefore we introduced the unsupervised learning module to help reduce the label

uncertainty as it performs clustering without considering the labels, and the labels are only used for the computation of the U-Score to measure the oncogenicity based on similarity. In this section, we conduct further analysis on our dataset by combining and comparing both S-Score and U-Score. Comparing S-Score and U-Score can help us to identify some suspicious mutations that might be labeled incorrectly.

6.2.1 Identifying Suspicious Mutations in COSMIC-FG1 v.57

Table XXI shows the possible S-Score and U-Score combinations for causative and non- causative instances with 0.5 as the threshold. The symbols ✓ and ✗ stand for expected and suspicious output, respectively. The instances labeled as causative in our COSMIC-FG1 dataset are based on the assumption that mutations which appear more than once in the COSMIC dataset are more likely to be causative than those appearing only once. Therefore instances that were labeled as causative are expected to have both high S-Score and high U-Score, while the commonly-occurring polymorphisms were labeled as non-causative because the polymorphisms were originally sampled from healthy individuals, and they are expected to have both low S-Score and low U-Score. Any other combinations marked as ✗ in Table XXI are suspicious and require further analysis.

Table XXI. Possible S-Score and U-Score Combinations for Causative and Non-Causative Instances

	Causative Label		Non-Causative Label	
	S-Score > 0.5	S-Score ≤ 0.5	S-Score > 0.5	S-Score ≤ 0.5
U-Score > 0.5	✓	✗	✗	✗
U-Score ≤ 0.5	✗	✗	✗	✓

✓= Expected, ✗= Suspicious

It was found that the majority of causative labeled instances and the majority of non-causative labeled instances fall into the "Expected" category states in Table XXI. Specifically, 219 out of 226 instances ($\approx 97\%$) labeled as causative fall into the "Expected" category, and 255 out of 331 instances ($\approx 77\%$) labeled as non-causative fall into the "Expected" category. This is a fairly good result to support our assumption that mutations which appear more than once in the COSMIC dataset are more likely to be causative than those appearing only once.

The suspicious labeled mutations in COSMIC-FG1 include 7 with causative label and 76 with non-causative label. Table XXII shows the detailed information of the 7 suspicious instances with causative labels, and Table XXIII describes the top 30 suspicious instances with non-causative label
.

Table XXII. Suspicious Mutations with Causative Labels in COSMIC-FG1 v.57

Rank	Gene	S-Score	U-Score	5S/5U	Position	WT	Mutant
1	BRAF	0.99683	0.49640	0.74662	605	S	N
2	EGFR	0.97213	0.49205	0.73209	717	V	A
3	ERBB2	0.93356	0.40680	0.67018	769	D	H
4	ALK	0.74793	0.44001	0.59397	1275	R	Q
5	MAP2K4	0.33680	0.64836	0.49258	154	R	W
6	MAP2K4	0.31930	0.57128	0.44529	184	S	L
7	FLT1	0.29884	0.51167	0.40526	943	E	K

Table XXIII. Top 30 Suspicious Mutations with Non-Causative Labels in COSMIC-FG1 v57

Rank	Gene	S-Score	U-Score	5S/5U	Position	WT	Mutant
1	ERBB3	0.87885	0.63776	0.75831	758	D	H
2	EGFR	0.88000	0.58050	0.73025	848	V	E
3	EGFR	0.65595	0.75921	0.70758	835	K	N
4	KDR	0.32547	0.78906	0.55726	848	V	E
5	EGFR	0.37216	0.68196	0.52706	962	R	G
6	EGFR	0.45902	0.57067	0.51485	952	V	I
7	ZAK	0.28244	0.73228	0.50736	115	G	S
8	EGFR	0.47082	0.50471	0.48777	890	H	Q
9	CSK	0.25772	0.66898	0.46335	357	S	G
10	PIM2	0.17315	0.74675	0.45995	165	K	R
11	AKT1	0.13271	0.75945	0.44608	319	E	G
12	DDR1	0.08366	0.77202	0.42784	835	R	W
13	ROR1	0.16061	0.69492	0.42776	566	T	M
14	TGFBR2	0.19817	0.63936	0.41876	316	E	V
15	TEC	0.25002	0.56572	0.40787	387	V	A
16	RET	0.14931	0.66529	0.40730	746	E	G
17	PAK4	0.05331	0.72528	0.38930	442	K	N
18	MOS	0.03661	0.73500	0.38581	221	V	A
19	TLK1	0.07387	0.68459	0.37923	646	D	V
20	NEK4	0.04907	0.70305	0.37606	64	N	D
21	MOS	0.00978	0.74150	0.37564	242	T	P
22	SCYL1	0.08409	0.66472	0.37440	59	Q	L
23	CDK4	0.00862	0.73671	0.37267	123	H	Q
24	RIPK3	0.09196	0.64979	0.37087	260	E	V
25	CDK5	0.00882	0.72408	0.36645	171	L	I
26	MAK	0.00663	0.72604	0.36634	269	L	F
27	IRAK1	0.12232	0.61005	0.36618	315	R	G
28	NLK	0.01709	0.70455	0.36082	208	I	T
29	MAP2K7	0.17266	0.54606	0.35936	259	L	F
30	MOS	0.00579	0.70674	0.35627	114	V	L

Mutations are sorting in descending order by the average of the S-Score and the U-Score.

References

[1] Robert C Holte. Very Simple Classification Rules Perform Well on Most Commonly Used Datasets. *Machine Learning*, 11(1):63–91, 1993.

[2] Kenji Kira and Larry A Rendell. A Practical Approach to Feature Selection. In *International Conference on Machine Learning*, pages 249–256, 1992.

[3] Ian H Witten, Eibe Frank, and Mark A Hall. *Data Mining: Practical Machine Learning Tools and Techniques*. Morgan Kaufmann, Amsterdam, 3 edition, 2011.

[4] M A Hall. *Correlation-based Feature Subset Selection for Machine Learning*. PhD thesis, Department of Computer Science, University of Waikato, 1999.

[5] Richard J Dobson, Patricia B Munroe, Mark J Caulfield, and Mansoor AS Saqi. Predicting deleterious nsSNPs: an analysis of sequence and structural attributes. *BMC Bioinformatics*, 7:217, January 2006.

[6] A. P. Dempster, N. M. Laird, and D. B. Rubin. Maximum likelihood from incomplete data via the em algorithm. *Journal of the Royal Statistical Society, Series B*, 39(1):1–38, 1977.

[7] Chuong B Do and Serafim Batzoglou. What is the expectation maximization algorithm? *Nature biotechnology*, 26(8):897–9, August 2008.

[8] Gilles Celeux and Gérard Govaert. A classification em algorithm for clustering and two stochastic versions. *Comput. Stat. Data Anal.*, 14(3):315–332, October 1992.
